# Systematic analysis of mitochondrial genes associated with hearing loss in the Japanese population: dHPLC reveals a new candidate mutation

**DOI:** 10.1186/1471-2350-12-135

**Published:** 2011-10-12

**Authors:** Hideki Mutai, Hiroko Kouike, Eiko Teruya, Ikuko Takahashi-Kodomari, Hiroki Kakishima, Hidenobu Taiji, Shin-ichi Usami, Torayuki Okuyama, Tatsuo Matsunaga

**Affiliations:** 1 Laboratory of Auditory Disorders, Division of Hearing and Balance Research, National Institute of Sensory Organs, National Tokyo Medical Center, Tokyo, Japan; 2 Department of Clinical Laboratory Medicine, National Center for Child Health and Development, Tokyo, Japan; 3 Division of Otorhinolaryngology, Department of Surgical Subspecialties, National Center for Child Health and Development, Tokyo, Japan; 4 Department of Otorhinolaryngology, Shinshu University School of Medicine, Nagano, Japan

## Abstract

**Background:**

Variants of mitochondrial DNA (mtDNA) have been evaluated for their association with hearing loss. Although ethnic background affects the spectrum of mtDNA variants, systematic mutational analysis of mtDNA in Japanese patients with hearing loss has not been reported.

**Methods:**

Using denaturing high-performance liquid chromatography combined with direct sequencing and cloning-sequencing, Japanese patients with prelingual (N = 54) or postlingual (N = 80) sensorineural hearing loss not having pathogenic mutations of m.1555A > G and m.3243A > G nor *GJB2 *were subjected to mutational analysis of mtDNA genes (*12S rRNA*, *tRNA*^*Leu(UUR)*^, *tRNA*^*Ser(UCN)*^, *tRNA*^*Lys*^, *tRNA*^*His*^, *tRNA*^*Ser(AGY)*^, and *tRNA*^*Glu*^).

**Results:**

We discovered 15 variants in *12S rRNA *and one homoplasmic m.7501A > G variant in *tRNA*^*Ser(UCN)*^; no variants were detected in the other genes. Two criteria, namely the low frequency in the controls and the high conservation among animals, selected the m.904C > T and the m.1105T > C variants in *12S rRNA *as candidate pathogenic mutations. Alterations in the secondary structures of the two variant transcripts as well as that of m.7501A > G in *tRNA*^*Ser(UCN) *^were predicted.

**Conclusions:**

The m.904C > T variant was found to be a new candidate mutation associated with hearing loss. The m.1105T > C variant is unlikely to be pathogenic. The pathogenicity of the homoplasmic m.7501T > A variant awaits further study.

## Background

Hearing loss manifests in more than 1 in 1000 persons at birth, and the frequency increases subsequently to 3 in 1000 by 4 years of age [1,2]. Approximately 50 to 70% of congenital and childhood deafness is estimated to be due to genetic mutations. In adults, the prevalence of hereditary hearing impairment has been estimated to be approximately 3.2 in 1000 [3]. Some of the mitochondrial DNA (mtDNA) genes, such as *12S rRNA*, *tRNA*^*Leu(UUR)*^, and *tRNA*^*Ser(UCN)*^, are known to be responsible for hereditary hearing loss [4]. Among them, the m.1555A > G mutation in *12S rRNA *is found relatively frequently (0.6-16%, depending on the ethnic group) in aminoglycoside-induced, congenital, and late-onset nonsyndromic hearing loss [4,5]. The m.1494C > T mutation in *12S rRNA *is also associated with aminoglycoside-induced and nonsyndromic hearing loss [6,7]. The m.3243A > G mutation in *tRNA*^*Leu(UUR) *^is associated with late-onset nonsyndromic hearing loss [8,9], maternally inherited diabetes and deafness (MIDD) [10,11], and mitochondrial myopathy, encephalopathy, lactic acidosis, stroke-like episodes (MELAS), which frequently presents with hearing loss [12,13]. The m.7445A > C/G/T [14-16], 7472insC [17], and 7510T > C mutations [18] in *tRNA*^*Ser(UCN) *^are also associated with aminoglycoside-induced, nonsyndromic, or syndromic hearing loss.

In addition, many other variants in *12S rRNA *have been proposed to be associated with hearing loss [4]. Some variants such as m.827A > G [19,20], 961T > C [21], 961delT + Cn [21,22], 1005T > C [22], and 1095T > C in *12S rRNA *[22-26] are not definitively related to hearing loss, because they have been found in subjects with normal hearing and/or are not conserved among mammals [19,27-30]. Moreover, a variety of mitochondrial haplogroups often localize in specific ethnic groups, making it difficult to determine whether the mtDNA variants are associated directly with diseases, indirectly as risk factors, or simply with rare subhaplogroups [31-34]. Accumulating reports of various novel mtDNA mutations associated with hearing loss prompted us to evaluate these variants in patients with hearing loss in Japan, where mtDNA mutation studies have focused on a few limited nucleotide positions [35,36].

A single cell contains hundreds of mitochondria, and the mtDNA in each mitochondrion is occasionally heterogeneous, a feature called heteroplasmy [37]. The proportion of pathogenic mutations of heteroplasmic mtDNA is considered to be one of the reasons for the wide range of severity of phenotypes seen in patients with mitochondrial-related diseases, such as those reported in the case of the m.3243A > G mutation [38-40]. Denaturing high-performance liquid chromatography (dHPLC) is a sensitive method to detect heteroplasmic mutations that can be overlooked by simple direct sequencing and comparison of the scanned peaks or restriction fragment length polymorphism-PCR [28,41]. In this study, we conducted a systematic mutational analysis of mtDNA by dHPLC combined with direct sequencing and cloning-sequencing in samples from Japanese patients with hearing loss.

## Methods

### Subjects

Subjects with bilateral sensorineural hearing loss were recruited by the National Tokyo Medical Center and collaborating hospitals. Subjects' medical histories were obtained and physical examinations were performed to exclude those subjects with syndromic symptoms, diseases of the outer or/and middle ear, and environmental factors related to hearing loss such as history of infectious diseases, premature birth, and newborn meningitis. Patients with a history of use of ototoxic drugs were included in the study because these drugs are known to be associated with mitochondrial hearing loss. Prior to this study, the patients were confirmed not to have the m.1555A > G and m.3243A > G mutations or not to be diagnosed as having *GJB2 *-caused hearing loss, as assessed by restriction fragment length polymorphism-PCR or together with direct sequencing if the heterozygotic 235delC mutation was detected in *GJB2 *[42,43]. The 134 subjects were classified into prelingual hearing loss (onset before 5 years old, 20 males and 34 females) or postlingual hearing loss (onset at 5 years old or later, 31 males and 49 females) [1]. The control group consisted of 137 unrelated Japanese individuals with normal hearing as examined by pure-tone audiometry. All subjects or their parents gave prior informed consent for participation in this study. This study was approved by the ethics committee of National Tokyo Medical Center.

### Screening for mtDNA mutations by dHPLC

DNA was extracted from blood samples using the Gentra Puregene DNA isolation kit (QIAGEN, Hamburg, Germany). Initially, whole mtDNA from each patient was amplified in three overlapping fragments (1351-8197, 6058-12770, and 11706-2258) [44] by LATaq DNA polymerase (TaKaRa BIO, Shiga, Japan). PCR was conducted at 94°C for 1 min followed by 30 cycles of 98°C for 10 s and 68°C for 6.5 min. Then, using the PCR products as templates, variants were analyzed by the Mitoscreen assay kit (Transgenomic, Glasgow, UK). We amplified the genes *12S rRNA*, *tRNA*^*Leu(UUR)*^, *tRNA*^*Ser(UCN)*^, *tRNA*^*Lys*^, *tRNA*^*His*^, *tRNA*^*Ser(AGY)*^, and *tRNA*^*Glu*^, for which mutations were reported to be associated with hearing loss on the Hereditary Hearing Loss Homepage [45] when the study was started. The PCR products using primer sets MT4 (for *12S rRNA*), MT6 (*tRNA*^*Leu(UUR)*^), MT10 (*tRNA*^*Ser(UCN)*^), MT11 (*tRNA*^*Lys*^), MT15 (*tRNA*^*His *^and *tRNA*^*Ser(AGY)*^), and MT18 (*tRNA*^*Glu*^) were incubated with the appropriate restriction enzymes, incubated for heteroduplex formation either with reference PCR products to detect homoplasmy or with their own PCR products to detect heteroplasmy, then analyzed by dHPLC (WAVE system, Transgenomic) according to the manufacturer's protocols.

The reference mtDNA was derived from a Japanese individual with normal hearing. Sequencing of the entire reference mtDNA revealed 750A > G and 1438A > G polymorphisms, and the mtDNA sequence was otherwise comparable to the revised Cambridge Reference sequence (AC_000021) [46,47]).

### DNA sequencing

When homoplasmic or heteroplasmic variants were detected, the PCR product was subjected to direct sequencing by the BigDye Terminator ver. 3 cycle sequencing kit and ABI genetic analyzer 3730 (Life Technologies, Carlsbad, CA). To sequence *12S rRNA*, an additional nested PCR product (656-1,266) was amplified with primers F (5'-tggtcctagcctttctattagctctt-3') and R (5'-tggcggtatataggctgagca-3'). To sequence *tRNA*^*Ser(UCN)*^, an additional nested PCR product (7,209-7,609) was amplified with primers F (5'-atgccccgacgttactcg-3') and R (5'- acctacttgcgctgcatgtg-3'). To determine the proportion of heteroplasmic 1005T > C variant in the *12S rRNA*, the nested PCR (656-1,266) product was cloned and sequenced. Nested PCR was carried out by replacing AmpliTaq Gold DNA polymerase with PrimeSTAR DNA polymerase, which has 3'-proofreading activity (TaKaRa BIO), followed by the Zero Blunt TOPO PCR cloning kit (Life Technologies). We sequenced 54 clones derived from the proband mtDNA and 24 clones derived from the mtDNA of each of five siblings. Sequencing data were analyzed by SeqScape ver2.6 (Life Technologies) and DNASIS Pro (Hitachisoft, Tokyo, Japan). The sequencing results for each patient were compared with the revised Cambridge Reference sequence to identify mtDNA variants. The uniqueness of each mutation was evaluated by comparison with the mtSNP database [48], MITOMAP [49], and the Uppsala mtDB database [50].

### Prediction of pathogenicity of mtDNA variants

The variants were evaluated based on double selection as proposed by Leveque and coworkers [51], with modification. Initially, we measured the frequencies of each variant found in the controls in our study (N = 137) and in the mtSNP database (N = 672, including: centenarians in Gifu, centenarians in Tokyo, type 2 diabetes mellitus patients (without or with vascular disorders), overweight young adult males, non-overweight young adult males, Parkinson's disease patients, and Alzheimer's disease patients in Japan). The variants with a frequency of more than 3% in one of the groups were considered as non-pathologic polymorphisms. We used a frequency threshold lower than that previously used (4%) [51] because the mtSNP database of Japanese individuals and the controls reflect the patient ethnic group background more closely than the mtDB and therefore requires a lower frequency threshold to exclude polymorphisms. The nucleotide conservation in each gene from human and 50 mammalian species was evaluated by ClustalW. The additional file lists the mammalian species and the accession numbers of the mtDNA (Additional File 1: Table S1). The variant frequencies in the mtDB were calculated to determine if the low variant frequencies measured in the controls reflect rare haplotypes in the Japanese population and are more common worldwide. All the variants were also analyzed with PhyloTree (mtDNA tree Build 10) [52] to search for previously characterized variants in haplogroups. Pathogenicity of the variants was also evaluated by predicting the secondary structures of the mitochondrial transcripts with or without the variant using Centroid Fold [53,54].

## Results

dHPLC screening and subsequent direct sequencing in the patients identified 12 homoplasmic or heteroplasmic variants in *12S rRNA *and 1 homoplasmic variant in *tRNA*^*Ser(UCN) *^(Table 1). In addition, the 3 homoplasmic variants, m.752C > T, 1009C > T, and 1107T > C in *12S rRNA *were detected in the controls by direct sequencing. All the patients and the controls appeared to have the non-pathogenic m.750A > G and 1438A > G variants, as previously noted [49]. No *tRNA*^*Glu*^, *tRNA*^*Leu(UUR)*^, *tRNA*^*Lys*^, *tRNA*^*His*^, or *tRNA*^*Ser(AGY) *^variants were detected. Table 1 lists the number of patients found with each variant, the frequencies of the variants in the controls and among Japanese individuals with various clinical conditions (mtSNP, N = 672), previous reports of the variants, and the frequencies of the variants in the mtDB. We evaluated two criteria, namely that the frequency of the variants be < 3% in both the controls and in the Japanese database (mtSNP) and that the variant nucleotide conserved by >50% among the 51 mammalian species we considered [51]; based on this analysis, two *12S rRNA *variants, m.904C > T and 1005T > C, were selected as candidate pathogenic mutations and subjected to further study. Although the homoplasmic m.7501T > A variant in *tRNA*^*Ser(UCN) *^did not meet the conservation criteria, it was also subjected to further study because several other *tRNA*^*Ser(UCN) *^mutations have been reported to be associated with hearing loss, whereas the m.7501T > A variant has not been studied for its pathogenicity.

**Table 1:** Mitochondrial DNA variants identified in this study

Gene	Mutation	Homo/heteroplasmy	prelingual HL (N = 54)	Late-onset HL (N = 80)	Controls (N = 137)	freq in controls (%)	Japanese **(N = 672)**^**a**^	freq in Japanese (%)	**conservation index**^**b**^	**Previous report**^**c**^	**mtDB**^**c **^M (N = 2704)	freq in mtDB (%)
*12S rRNA*	663A > G	homoplasmy	3	5	2	**1.5**	48	7.1	**29/51**	yes	86	3.2
	709G > A	homoplasmy	7	7	12	8.8	125	18.6	19/51	yes	444	16.4
	750A > G	homoplasmy	54	80	137	100.0	no data	no data	**49/51**	yes	2682	96.7
	752C > T	homoplasmy	0	0	9	6.6	17	2.5	44/51	yes	20	0.7
	827A > G	homoplasmy	4	3	3	**2.2**	25	3.7	**48/51**	yes	54	**2.0**
	**904C > T**	homoplasmy	1	0	0	**0.0**	0	**0.0**	**48/51**	none	0	**0.0**
	961insC	homoplasmy	1	0	3	**2.2**	1	**0.1**	9/51	yes	37	**2.0**
	961delT+ Cn	both	0	1	4(2)^d^	**2.9**	no data	no data	9/51	yes	no data	no data
	**1005T > C**	both	1	1(1)	1	**0.7**	1	**0.1**	**33/51**	yes	7	**0.3**
	1009C > T	homoplasmy	0	0	1	0.7	1	0.1	9/51	yes	2	0.1
	1041A > G	homoplasmy	0	4	5	3.6	11	**1.6**	**26/51**	yes	14	**0.5**
	1107T > C	homoplasmy	0	0	6	4.4	29	4.3	30/51	yes	34	1.26
	1119T > C	homoplasmy	1	2	7	5.1	20	3.0	20/51	yes	26	1.0
	1382A > C	homoplasmy	0	1	11	8.0	62	9.2	**38/51**	yes	65	**2.4**
	1438A > G	homoplasmy	54	80	137	100.0	662	98.5	**46/51**	yes	2620	96.9
*tRNA*^*Ser(UCN)*^	7501T > A	homoplasmy	0	3	0	**0.0**	1	**0.1**	15/51	yes	1	**0.0**

Mitochondrial gene variants that met the criterion for association with hearing loss (HL) are underlined and in bold type. ^a^Data from the mtSNP database [48]. ^b^Based on the results of the multiple alignment by ClustalW. See Additional File 1: Table S1 for information on the species used to calculate the sequence conservation. ^c^Uppsala mtDB database [50]. ^d^Each number in parentheses indicates the number of individuals with a heteroplasmic variant.

**Figure 1:**
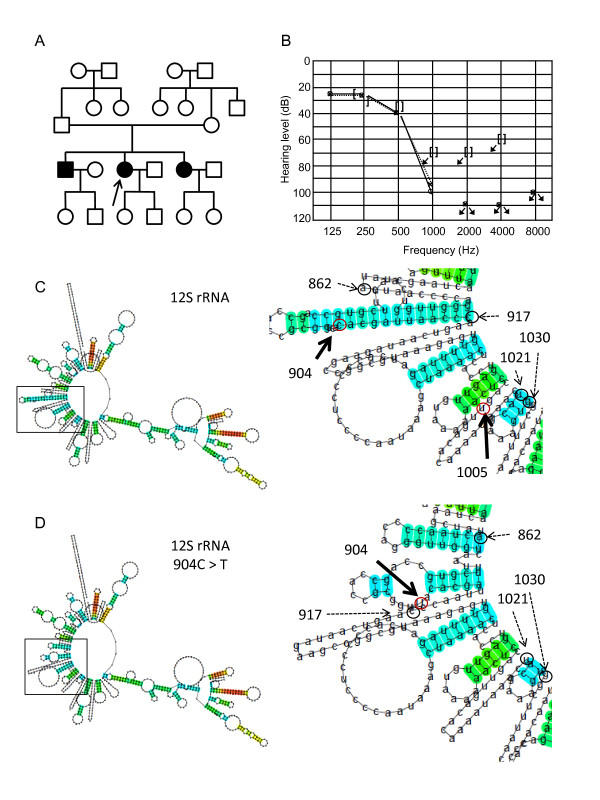
**Pedigree of a family carrying the m.904C > T variant**. (A) Pedigree of a family carrying the homoplasmic m.904C > T variant. Individuals with hearing loss are indicated by filled symbols. The arrow indicates the proband. (B) Audiogram of the proband of m.904C > T. Open circles with the line indicate the air conduction thresholds of the right ear; the X's with dotted line indicate the air conduction thresholds of the left ear; [, bone conduction thresholds of the right ear; ], bone conduction thresholds of the left ear. Arrows indicate the scale-out level of hearing loss. (C, D) Secondary structures of wild-type 12S rRNA (C) and 12S rRNA with the m.904C > T (D) predicted by Centroid Fold. To the right is shown an enlargement of the region of predicted secondary structures surrounding nucleotide positions including 904 and 1005 (bold arrows with red circles). Positions 862, 917, 1021, and 1030 are marked by dashed arrows with black circles for easy comparison of the structural changes. Each predicted base pair is indicated by a gradation of color (red to blue) corresponding to the base-pairing probability from 1 (red) to 0 (blue) according to Centroid Fold.

A novel homoplasmic m.904C > T variant in the*12S rRNA *was found in a 46-year-old female patient (Figure 1A). She did not possess additional mtDNA pathogenic mutations and showed prelingual, progressive hearing loss with tinnitus. The patient was suspected of hearing impairment as early as 4 years old and was diagnosed with sensorineural hearing loss at age 11. The audiometric examination showed mild hearing loss at low frequencies and no response at 1 kHz and higher frequencies (Figure 1B). She had no response to an otoacoustic emission test, indicating dysfunction of the auditory outer hair cells. The patient had no history of treatment with ototoxic drugs and did not suffer from any other symptoms. The siblings also suffered from prelingual, severe hearing loss (with similar ages of onset and severity), but their parents had normal hearing (Figure 1A). The patient bore two children with normal hearing. DNA samples were not obtained from other family members. The secondary structure of the variant 12S rRNA predicted by Centroid Fold suggested that substitution of C > T (transcribed as U) at position 904 of the *12S rRNA *results in gross structural alteration of the transcript region that includes nucleotide positions 862 to 917, in addition to truncation of the stem-like structure from positions 1021 to 1030 (Figure 1C and 1D), implicating a significant role for 904C in 12S rRNA folding.

**Figure 2:**
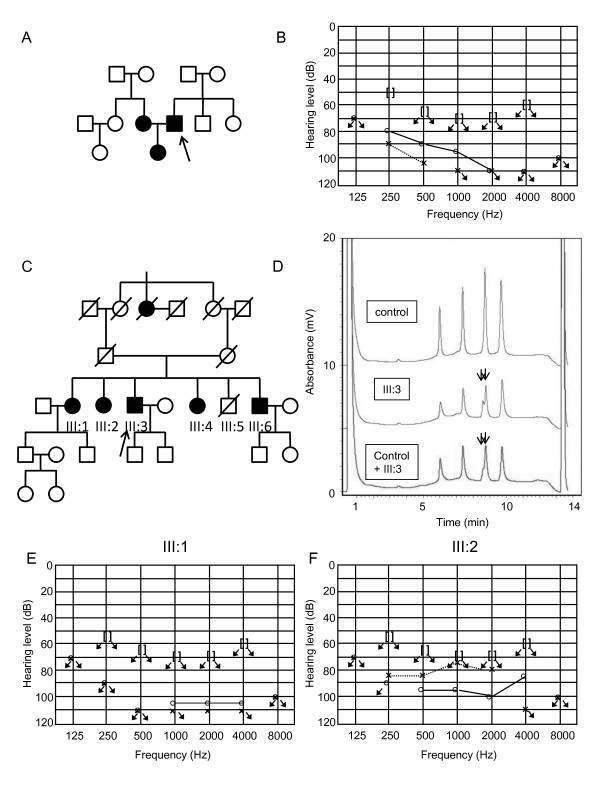
**Pedigrees of families carrying the m.1005T > C variant**. (A,B) Pedigree of a family carrying the homoplasmic m.1005T > C (A), and the audiogram of the proband (B). (C-F) Pedigree of a family carrying heteroplasmic m.1005T > C (C), and the chromatogram of dHPLC of the MT4 fragment of the proband (D). The arrows indicate split peaks of the fragment owing to the heteroplasmic m.1005T > C. Audiograms of the siblings (III:1, 2) are shown in (E-F).

The homoplasmic m.1005T > C variant in the *12S rRNA *was found in a male patient with prelingual, severe hearing loss (Figure 2A, B). The patient's spouse had prelingual hearing loss owing to measles, and their child also had prelingual hearing loss. The m.1005T > C variant was not detected in the patient's spouse or daughter. DNA samples were not obtained from other family members.

**Figure 3:**
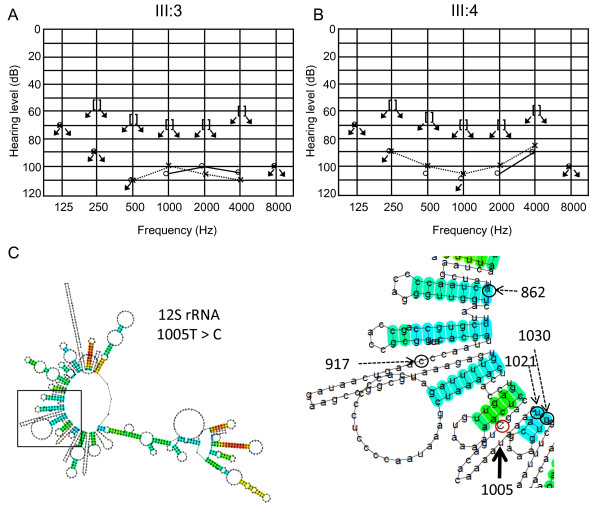
**Pedigrees of families carrying the m.1005T > C variant (continued)**. (A,B) Audiograms of the siblings (III: 3, 4) of a family carrying the heteroplasmic m.1005T > C (Figure 2C). (C) Predicted secondary structure of the *12S rRNA *transcript with the m.1005T > C. To the right is shown an enlargement of the region of predicted secondary structures surrounding nucleotide position 1005.

The heteroplasmic m.1005T > C variant together with the homoplasmic mutation m.709G > A was detected in a male patient from a consanguineous marriage of parents with normal hearing (Figure 2C). In the proband (III:3), onset of hearing loss and diabetes mellitus occurred in his 40s. Among his five siblings, four (III:1, 2, 4, 6) also showed adult-onset hearing loss between age 20 and 50 years, but they did not have diabetes mellitus. The fifth sibling suffered from infantile paralysis and died at age 6 (III:5). Cloning of the fragment of *12S rRNA*, which demonstrated apparent heteroduplex formation (Figure 2D, arrow), yielded 12 of 54 clones (22%) with the m.1005T > C variant. However, the m.1005T > C variant was not detected in 24 clones derived from the mtDNA from each of these siblings, indicating that the variant was absent in the siblings or the frequency was less than 4%. The audiograms showed severe to profound hearing loss in the siblings III:1, 2, 3, and 4 (Figure 2E, F, 3A, B). The secondary structure of the *12S rRNA *variant predicted by Centroid Fold indicated that the m.1005T > C induces a gross structural alteration in the transcript, including nucleotide positions 862 to 917 (Figure 1C and 3C).

**Figure 4:**
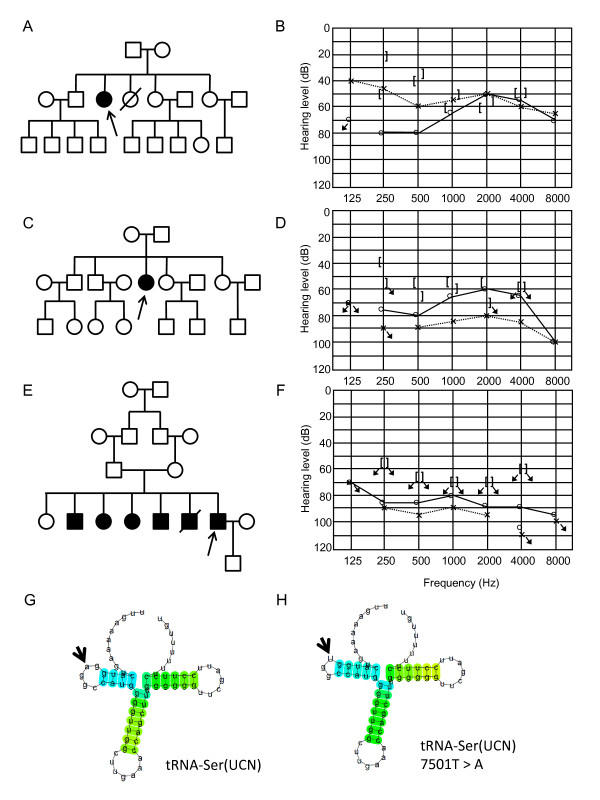
**Pedigrees of families carrying the m.7501T > A variant**. (A-F) Pedigrees of three families carrying the homoplasmic m.7501T > A, and audiograms of the probands (A and B, C and D, E and F). (G,H) Predicted secondary structure of the *tRNA*^*Ser(UCN) *^transcript (G) and the tRNA^Ser(UCN) ^with m.7501T > A (H). Because the gene is transcribed in the reverse direction, thymine at 7501 (G) and adenine (H) are indicated as a and u, respectively (bold arrows).

Three patients appeared to carry the homoplasmic m.7501T > A variant in *tRNA*^*Ser(UCN) *^(Figure 4A, C, E). One female patient suffered from episodic vertigo from age 27 years followed by tinnitus and fluctuant, moderate progressive hearing loss, and she had no familial history of hearing loss (Figure 4A, B). Another female patient suffered from tinnitus beginning at age 24 years and had been exposed to streptomycin from age 36 to 37 for treatment of tuberculosis (Figure 4C, D). She suffered from fluctuant, moderate hearing loss from her 50s and had no familial history of hearing loss. The third patient was a male from a consanguineous marriage of parents with normal hearing and showed non-progressive, severe hearing loss from childhood without tinnitus or vertigo (Figure 4E, F). Later, he was also found to have X-linked spinal and bulbar muscular atrophy (SBMA/Kennedy-Alter-Sung disease/Kennedy's disease). In this family, six of seven siblings showed hearing loss. Family members other than the proband did not participate in this study. According to the secondary structure prediction by Centroid Fold, the m.7501T > A in *tRNA*^*Ser(UCN) *^(which is transcribed as U in the reverse direction) causes an elongation of the D-arm in the transcript by reducing the size of the D-loop of tRNA^Ser(UCN) ^(Figure 4G, H), which might affect biosynthesis of mitochondrial proteins [55].

## Discussion

In our study, screening of mtDNA by dHPLC and direct sequencing detected 15 variants in *12S rRNA *and 1 variant in *tRNA*^*Ser(UCN)*^. Comparison of the variant frequencies in controls, assessment of nucleotide conservation among mammalian species, and structural analysis of the transcript was used to select candidate mutations associated with hearing loss. No variants in *tRNA*^*Leu(UUR)*^, *tRNA*^*Lys*^, *tRNA*^*His*^, *tRNA*^*Ser(AGY)*^, or *tRNA*^*Glu *^were detected in the subjects studied here, suggesting that the mutations in these genes associated with hearing loss are not common in the Japanese population.

To our knowledge, the homoplasmic m.904C > T variant in *12S rRNA *has not been reported elsewhere. Lack of symptoms in the maternal relatives does not exclude mitochondrial transmission, because penetrance of *12S rRNA *mutations can be extremely low, as seen in the m.1555A > G associated with hearing loss [56]. Conservation of the nucleotides among mammals and gross alteration of the predicted secondary structure of the *12S rRNA *transcript suggest that the m.904C > T variant might affect auditory function by changing the efficiency with which mRNAs are transcribed to yield mitochondrial proteins.

A patient with the homoplasmic m.1005T > C variant in the *12S rRNA *had a child with prelingual hearing loss. The inheritance of hearing loss in the child is likely due to the transmission of an autosomal mutation, not mtDNA, from the male proband. Therefore, the data for this family may not provide unequivocal information about the pathogenicity of the m.1005T > C variant [4,22,27,30].

Identification of the heteroplasmic m.1005T > C variant in a patient with hearing loss is a novel finding, because this variant has been known only as homoplasmic [22,27,30,34]. We did not verify that the heteroplasmic m.1005T > C variant was correlated with hearing loss because four of five siblings of the proband had hearing loss without carrying the variant, whereas it might be associated with diabetes mellitus. However, it is difficult to exclude the possibility of association of the heteroplasmic variant detected in blood samples with mitochondrial diseases such as deafness. Frequencies of heteroplasmy of mtDNA vary considerably among tissues in the same individual (for instance, [37,57,58]). Therefore, it is possible that the frequency of the m.1005T > C variant in the inner ear cells of the siblings is much higher than in the blood cells and thus may underlie the hearing loss.

Another finding in this study is that three patients with postlingual hearing loss had the homoplasmic m.7501T > A variant in *tRNA*^*Ser (UCN)*^. Various mutations in *tRNA*^*Ser(UCN)*^, such as m.7445A > G [15,16], 7472insC [17,59], 7505T > C [60], 7510T > C [18], and 7511T > C [51,59,61], are associated with various types of hearing loss (syndromic or nonsyndromic, prelingual or late-onset), raising the possibility that the m.7501T > A variant, reported elsewhere without detailed investigation [33], is also associated with hearing loss. The low conservation of the variation at this position (29% among mammals) does not support the pathogenicity of the variant, in contrast to the much higher conservation at m.7472A (61%), 7505A (98%), 7510T (78%), and 7511T (98%). On the other hand, the m.7501T > A variant is predicted to modify the secondary structure of the D-arm in the *tRNA*^*Ser(UCN) *^transcript; the D-arm is important for the stability of the transcript and the general rate of mitochondrial protein synthesis [55]. Further investigation, such as haplogroup analysis or generating lymphoblastoid cell lines to measure endogenous respiration rates, may help to define the pathogenicity of the m.7501T > A variant.

All other variants found in this study, such as m.827A > G, 961insC, and 961delT + Cn, which have been discussed elsewhere with respect to their pathogenicity [21,22,27,30,62], were considered to be non-pathologic polymorphisms because they were found frequently in the controls. The other variants, m.663A > G, 709G > A, 750A > G, 752C > T, 1009C > T, 1041A > G, 1107T > C, 1119T > C, 1382A > C, and 1438A > G, were frequently detected in the controls and considered to be nonpathogenic polymorphisms, which is in consistent with a previous report [27]. The spectrum of variants of mitochondrial genes in Japanese individuals was similar to that in a Chinese population [27], for which most of the variants detected in this study (other than the m.904C > T and 7501T > A) have been reported. In contrast, the spectrum was dissimilar to those in other ethnic groups such as the Polish population [19,63]. Our results indicate that ethnic background should be taken into consideration when studying the pathogenicity of mtDNA variants based on their frequencies in controls.

## Conclusions

We sought to detect mitochondrial variants other than m.1555A > G or 3243A > G mutations, which are known to be related to hearing loss, by dHPLC, direct sequencing, and cloning-sequencing in samples from Japanese patients with hearing loss. The homoplasmic m.904C > T variant in *12S rRNA *was considered to be a new candidate mutation associated with hearing loss. The pathogenicity of the m.7501T > A variant in *tRNA*^*Ser(UCN) *^remains inconclusive, and other variants identified in this study, including the heteroplasmic m.1005T > C variant, are not positively associated with hearing loss. No variants were detected in the in *tRNA*^*Leu(UUR)*^, *tRNA*^*Lys*^, *tRNA*^*His*^, *tRNA*^*Ser(AGY)*^, and *tRNA*^*Glu*^.

## Competing interests

The authors declare that they have no competing interests.

## Authors' contributions

HM participated in cloning and sequencing, data analysis, and drafted the manuscript. HKo, ET, ITK, and HKa established and conducted the dHPLC analysis, sequencing, and data analysis. HT, SU, and TO coordinated the study and helped with gene analysis. TM planned and organized the study, examined patients, analyzed data, and helped draft the manuscript. All the authors read and approved the final manuscript.

## Pre-publication history

The pre-publication history for this paper can be accessed here:

http://www.biomedcentral.com/1471-2350/12/135/prepub

## Supplementary Material

Additional file 1 **Table S1**. List of animal species and the accession numbers of the mtDNA (GenBank) used to calculate nucleotide conservation. Click here for file
