# Supplementary material for: Systematic analysis of mitochondrial genes associated with hearing loss in the Japanese population: dHPLC reveals a new candidate mutation
Source: BMC Med Genet. 2011 Oct 12;12:135. doi: 10.1186/1471-2350-12-135 (PMC3207971; doi:10.1186/1471-2350-12-135)
Supplement: Additional file 1 — Table S1. List of animal species and the accession numbers of the mtDNA (GenBank) used to calculate nucleotide conservation. [file 1471-2350-12-135-S1.DOC]

| human | AC_000021 |
| --- | --- |
| pygmy chimpanzee | NC_001644 |
| lowland gorilla | NC_011120 |
| crab-eating monkey | NC_012670 |
| aye-aye | NC_010299 |
| ring-tailed lemur | NC_004025 |
| mouse | NC_005089 |
| rat | NC_001665 |
| earth-colored mouse | NC_010650 |
| jerboa | NC_005314 |
| mole rat | NC_005315 |
| gymnure | NC_010298 |
| cow | NC_006853 |
| goat | NC_012096 |
| goral | NC_013751 |
| sheep | NC_001941 |
| red deer | NC_007704 |
| reindeer | NC_007703 |
| giraffe | NC_012100 |
| vicugna | NC_013558 |
| hippopotamus | NC_000889 |
| pig | NC_000845 |
| bowhead whale | NC_005268 |
| grey whale | NC_005270 |
| boutu | NC_005276 |
| franciscana | NC_005277 |
| narwhal | NC_005279 |
| bearded seal | NC_008426 |
| Australian sea lion | NC_008419 |
| California sea lion | NC_008416 |
| crab-eater seal | NC_008423 |
| dog | NC_002008 |
| red fox | NC_008434 |
| cave bear | NC_011112 |
| polar bear | NC_003428 |
| giant panda | NC_009492 |
| lesser panda | NC_011124 |
| badger | NC_011125 |
| wolverine | NC_009685 |
| skunk | NC_010497 |
| bobcat | NC_014456 |
| horse | NC_001640 |
| hyrax | NC_010301 |
| American pika | NC_005358 |
| European hare | NC_004028 |
| Asiatic elephant | NC_005129 |
| pangolin | NC_004027 |
| tamandua | NC_004032 |
| Japanese mole | NC_005035 |
| banded-hare wallaby | NC_008447 |
| common ring-tailed opossum | NC_006519 |

**Table S1**
